# Metabolic Alterations in Mothers Living with HIV and Their HIV-Exposed, Uninfected Infants

**DOI:** 10.3390/v16020313

**Published:** 2024-02-19

**Authors:** Louise D. V. du Toit, Shayne Mason, Mari van Reenen, Theresa M. Rossouw, Roan Louw

**Affiliations:** 1 Department of Immunology, Faculty of Health Sciences, University of Pretoria, Pretoria 0001, South Africa; theresa.rossouw@up.ac.za; 2 UP Research Centre for Maternal, Fetal, Newborn and Child Health Care Strategies, University of Pretoria, Pretoria 0001, South Africa; 3 Maternal and Infant Health Care Strategies Research Unit, South African Medical Research Council, Pretoria 0001, South Africa; 4 Human Metabolomics, Faculty of Natural and Agricultural Sciences, North-West University, Potchefstroom 2520, South Africa; shayne.mason@nwu.ac.za (S.M.); 12791733@nwu.ac.za (M.v.R.); roan.louw@nwu.ac.za (R.L.)

**Keywords:** HIV-exposed uninfected, HEU, metabolomics, profile, infant health, NMR

## Abstract

HIV-exposed, uninfected (HEU) children present with suboptimal growth and a greater susceptibility to infection in early life when compared to HIV-unexposed, uninfected (HUU) children. The reasons for these findings are poorly understood. We used a metabolomics approach to investigate the metabolic differences between pregnant women living with HIV (PWLWH) and their HEU infants compared to the uninfected and unexposed controls. Untargeted metabolomic profiling was performed using ^1^H-NMR spectroscopy on maternal plasma at 28 weeks’ gestation and infant plasma at birth, 6/10 weeks, and 6 months. PWLWH were older but, apart from a larger 28 week mid-upper-arm circumference, anthropometrically similar to the controls. At all the time points, HEU infants had a significantly reduced growth compared to HUU infants. PWLWH had lower plasma 3-hydroxybutyric acid, acetoacetic acid, and acetic acid levels. In infants at birth, threonine and myo-inositol levels were lower in the HEU group while formic acid levels were higher. At 6/10 weeks, betaine and tyrosine levels were lower in the HEU group. Finally, at six months, 3-hydroxyisobutyric acid levels were lower while glycine levels were higher in the HEU infants. The NMR analysis has provided preliminary information indicating differences between HEU and HUU infants’ plasma metabolites involved in energy utilization, growth, and protection from infection.

## 1. Introduction

The successful prevention of vertical transmission between pregnant women living with HIV (PWLWH)/mothers living with HIV (MLWH) and their infants has increased the number of infants who are HIV-exposed but uninfected (HEU) [1,2]. In South Africa, approximately 30% of newborns are HEU and have increased morbidity and mortality rates compared to infants born to HIV-uninfected mothers [3].

As part of the prevention of vertical transmission, the introduction of potent antiretroviral therapy (ART) during pregnancy and breastfeeding has improved the health of PWLWH and MLWH [2]. Despite the success of ART, immune dysfunction, inflammation, and metabolic abnormalities persist in people living with HIV (PLWH) on ART, predicting their subsequent morbidity and mortality [2].

HIV infection has major effects on maternal physiology and pregnancy outcomes [4]. However, it remains unclear whether the inflammatory and metabolic derangements in PWLWH on ART and/or potential ART toxicity affect infant development in the millions of HEU infants born globally each year. Immunometabolic alterations could set infants on a path towards suboptimal growth, as well as a lifelong compromised immune function [5,6,7,8].

Metabolomics is the identification and quantification of small metabolites (the metabolome) and is frequently applied in the assessment of metabolic alterations, caused by diseases or treatments, in biological systems [9,10]. Proton nuclear magnetic resonance (^1^H-NMR) spectroscopy is an untargeted metabolomics technique, which allows for the rapid and highly discriminatory identification and quantification of a wide range of compounds [11,12]. It is an attractive approach since it requires little sample preparation, and is non-destructive, reproducible, and unbiased [12,13].

In this study, we characterized the metabolomic profiles of PWLWH and their HEU infants and compared them to the profiles of HIV-uninfected mothers and their HIV-unexposed, uninfected (HUU) infants.

## 2. Materials and Methods

This study formed part of a larger study, the Siyakhula study [14], for which ethics approval by the Research Ethics Committee of the Faculty of Health Sciences of the University of Pretoria had been obtained (protocol number 294/2017). The larger study recruited 315 women, 152 PWLWH and 163 HIV-uninfected pregnant women, from antenatal clinics in Southwest Tshwane, South Africa. Participant recruitment and sample collection commenced on 1 December 2017 and concluded on 16 June 2023. Recruitment was conducted before 22 weeks’ gestation, with the subsequent follow-up of the mother–infant pairs throughout pregnancy until 24 months post-partum. Women received specialized antenatal and obstetric care through the Maternal and Infant Health Care Strategies Unit (MIHCSU) of the South African Medical Research Council (MRC). Written informed consent was obtained from the women before study inclusion. Subsequently, they received three ultrasound examinations during pregnancy, with the first ultrasound examination performed at Kalafong Tertiary Academic Hospital at 22 weeks’ gestation. The gestational age was verified using the second-trimester biometry.

Exclusion criteria included the inability to obtain informed consent, maternal hypertension, diabetes mellitus or other serious pre-existing medical disorders, infection with *Mycobacterium tuberculosis*, multiple pregnancies, and fetuses with chromosomal or structural abnormalities.

### 2.1. Metabolomics Sub-Study

Ethics approval for the metabolomics sub-study was obtained from the Research Ethics Committee of the Faculty of Health Sciences of the University of Pretoria (610/2019). Samples were selected from the larger Siyakhula study and excluded based on the following criteria: (1) no birth weight or length available, (2) use of corticosteroids during pregnancy, (3) maternal HIV status uncertain, (4) maternal ART regimen unknown, (5) absence of maternal oral glucose tolerance test during pregnancy, (6) maternal age >45 years during pregnancy, and (7) gestational age at birth <35 weeks.

We performed metabolomic analysis on samples from the mothers at 28 weeks’ gestation. We analyzed infant samples at birth, as well as at 6 or 10 weeks, and 6 months post-partum. These time points were selected based on the expected infant feeding practices at these points. At birth, we aimed to have a clear view of the metabolic profile as affected by only the intrauterine environment. At 6 or 10 weeks, infants were likely to be exclusively breast- or formula-fed and at 6 months, infants would have started on a mixed diet of breast or formula milk and solids.

At the 28 week antenatal visit, 15–30 mL of blood was collected from the mothers. At birth, cord blood was obtained, and 1–5 mL of blood was collected from the infants at each of the post-partum time points specified. At each study visit, anthropomorphic and clinical data were captured on standardized forms. To maximize the number of infants available, the 6 and 10 week blood samples were combined into a single time point.

### 2.2. Demographic and Anthropometric Data

Maternal and infant demographic and anthropometric data were captured at each study visit. For PWLWH, this included age, CD4+ T-cell counts, HIV viral loads, weight, height, body mass index (BMI), and mid-upper-arm circumference (MUAC). For the infants, these data included method of delivery, weight, length, BMI, head circumference (HC), and MUAC, as well as corresponding z-scores and breastfeeding status.

Z-scores express the number of standard deviations (SDs) above or below the reference mean or median value for an anthropometric variable in a specific population, and are used in pediatric medicine to accurately assess the growth and nutritional status [15]. For instance, a weight-for-age z-score (WAZ) compares an infant’s weight to the weight of an infant of the same age and sex, and is used to detect the presence of malnutrition or disease [16]. Moderate malnutrition is defined as a WAZ between −3 and −2 SDs below the mean of the WHO child growth standards. Similarly, moderate wasting (low weight-for-height [WHZ]) and stunting (low length-for-age [LAZ]) are defined as z-scores between −3 and −2 SDs. Z-score values below −3 indicate severe wasting and stunting [17].

### 2.3. Experimental Procedures

#### 2.3.1. Sample Processing

Whole blood collected in tubes (Becton, Dickinson and Company, Franklin Lakes, NJ, USA) containing the anticoagulant EDTA was used. Plasma was isolated from the whole blood by centrifuging (Beckman Coulter, Brea, CA, USA) at 335× *g* for 10 min at 22 °C. The plasma was collected into a 15 mL centrifuge tube (Sigma-Aldrich, St. Louis, MO, USA) and centrifuged (Beckman Coulter) at 750× *g* for 10 min at 22 °C. This was carried out to remove platelets and residual red blood cells. Plasma aliquots were frozen (Thermo Fisher, Waltham, MA, USA) at −80 °C until laboratory analyses were performed. Untargeted metabolomic profiling was performed using ^1^H NMR spectroscopy as described by Mason et al. [18]. Briefly, proteins were removed from the plasma samples using 10 kDa centrifugal filters (Sigma-Aldrich), and 54 µL of the filtrate was added to 6 µL buffer solution (potassium phosphate buffer at pH 7.4, in deuterium oxide, with the internal standard trimethylsilylpropanoic acid). The 60 µL sample was transferred to a 2 mm glass NMR tube (Sigma-Aldrich) and analyzed in a 500 MHz NMR spectrometer (Bruker, Billerica, MA, USA) at 300 K, using NOESY water pre-saturation, a spectral width of 6000, a receiver gain of 64, and 128 scans of an 8 µs pulse and a 4 s delay. Processing was carried out using Bruker Topspin (V3.6.4).

#### 2.3.2. Statistical Analyses

Variable-sized binning was performed to avoid splitting peaks across multiple bins and to remove regions of spectral noise. Significant bins were selected using various statistical methods. The metabolites used for interpretation were based on concentrations calculated after peak identification based upon pure compound spectra libraries. The following statistical methods were applied to the data: (1) Mann–Whitney test (*p*-value < 0.05) [19]; (2) effect size (medium (d > 0.6)/large (d > 0.8)). The probability of superiority (Aw) of effect size measure was selected. It improves the accessibility of information, i.e., the measure is easily interpreted as the probability that a case randomly selected from group 1 will have a higher value than a case randomly selected from group 2. Aw is robust to unequal sample sizes and does not make any assumptions regarding normality or homogeneity of variances. The R package RProbSup, https://cran.r-project.org/package=RProbSup (accessed on 23 February 2022), was used to compute Aw, given default settings. Compounds with an Aw value exceeding 0.64 between groups were shortlisted as informative. An Aw value of 0.64 corresponds to a moderate effect [20]; (3) XERp (100% selected and an accuracy ≥ 60%). XERp is a nonparametric univariate method that minimizes classification error rates, supported by statistical significance or *p*-values, to flag variables with a predictive potential. XERp applies the leave-one-out validation to shortlist variables that consistently produce statistically significantly low predictive error rates. XERp error rates are deconstructed into sensitivity and specificity percentages and, if both are acceptable, the variable is considered informative [21]. (4) Finally, a two-way ANOVA interaction *p* (unadjusted) of ≤0.05 is considered to supplement univariate findings [19].

Growth data were analyzed in Stata 17 (StataCorp, College Station, TX, USA). After normality testing, growth parameters and metabolites were described as mean ± standard deviation (SD) or median and interquartile range (IQR), as appropriate. Categorical variables were described as proportions. Continuous variables were compared between groups with the Student’s *t*-test or Kruskal–Wallis test and categorical variables by means of Pearson’s chi-square or Fisher’s exact test, as appropriate.

## 3. Results

Sixty participants of African descent and their infants were included in this study. Of these, 29 were PWLWH and were on ART consisting of a combination of two nucleos(t)ide reverse transcriptase inhibitors (N(t)RTI), tenofovir disoproxil fumarate (TDF) and emtricitabine (FTC), and a non-nucleoside reverse transcriptase inhibitor (NNRTI), efavirenz (EFV). All HEU infants were treated according to the South African Guideline for the Prevention of Mother to Child Transmission of Communicable Infections of 2019 [22]. Accordingly, all low-risk infants (*n* = 21) received nevirapine for six weeks and all high-risk infants (maternal HIV viral load >1000 copies/mL or on ART for <4 weeks prior to delivery; *n* = 8) received nevirapine together with six weeks of zidovudine.

All women had a normal oral glucose tolerance test result approximately four weeks before the 28 week visit. PWLWH were significantly older than uninfected pregnant women (Table 1). While no significant differences were observed between the two groups regarding weight, height, or BMI at 28 weeks’ gestation, PWLWH had a larger MUAC. Significantly more PWLWH met the definition of obesity during late pregnancy (i.e., MUAC > 30.5 cm): 12/28 (42.9%) versus 5/31 (16.1%), *p* = 0.024. This difference was no longer evident at the time of delivery. A larger proportion of MLWH had a normal vaginal delivery when compared to uninfected women, but the difference was not statistically significant.

The gender distribution was similar between the HEU and HUU infants: HEU 17 (58.6%) male versus HUU 15 (48.4%) male; *p* = 0.4270. The mean gestation age was 38.8 ± 1.3 weeks and there was no difference between the two groups (HEU 38.6 ± 1.4 versus HUU 38.9 ± 1.2; *p* = 0.3305).

Of the HEU infants, 5/29 (17.2%) had a low birth weight (<2500 g), while 2/31 (6.5%) of the HUU infants had a low birth weight, but this difference was not statistically significant (*p* = 0.247). However, HEU infants had a lower BMI at birth, 6 weeks, and 10 weeks; a lower weight at 6 weeks; a lower weight-for-length z-score (WHZ) and BMI-for-age z-score (BAZ) at 6 and 10 weeks; and a lower MUAC and MUAC z-score (MUACZ) at 6 months. Significantly more HIV-uninfected mothers were breastfeeding their infants at 6 and 10 weeks than MLWH (Table 2 and Table 3). A sensitivity analysis of infants who were exclusively breastfed showed a similar pattern in terms of the anthropometric parameters (Supplementary Table S1).

Metabolomics data for all the identified metabolites are shown in Supplementary Tables S2–S5. Significant differences were observed in metabolite concentrations for PWLWH vs. uninfected women at 28 weeks’ gestation. Among the PWLWH, 3-hydroxybutyric acid, acetoacetic acid, and acetic acid levels were lower than in the HIV-uninfected pregnant women. Significant differences were also observed in metabolite concentrations between HEU and HUU infants at all three time points. In infants at birth, threonine and myo-inositol levels were lower in the HEU group and formic acid levels were higher. At 6/10 weeks, betaine and tyrosine levels were lower in the HEU group. Finally, at 6 months, 3-hydroxyisobutyric acid levels were lower while glycine levels were higher in the HEU infants (Table 4).

Excluding infants not being breastfed at 6/10 weeks, the results revealed similar differences for betaine (*p* = 0.0453) and tyrosine (*p* = 0.0431) levels, but also differences in glycine levels (HEU 332.3 [IQR 290.2–362.8] versus HUU 283.9 [IQR 239.3–348.0]; *p* = 0.0315). Excluding infants not being breastfed at 6 months, the results did not alter the difference in glycine levels between the groups (*p* = 0.0205), but 3-hydroxyisobutyric acid levels were no longer significantly different (*p* = 0.4643).

## 4. Discussion

### 4.1. Clinical Parameters

Maternal nutritional status, as estimated by various anthropometric parameters, is associated with fetal growth and birth weight [23]. The relationship between MUAC and birth weight has been confirmed in populations living both with or without HIV [23,24]. PWLWH in our cohort had a larger mean MUAC at 28 weeks’ gestation, and a higher proportion with a MUAC indicating an overweight nutritional status [25]. Maternal anthropometry therefore does not explain the poorer growth seen in the HEU infants at all time points. PWLWH were also significantly older than their HIV-uninfected counterparts. This is potentially due to the fact that HIV affects fertility, leading to a delay in becoming pregnant [26].

In this cohort, HEU infants had significantly poorer growth compared to HUU infants of the same socioeconomic background. Our findings are consistent with another study in South Africa that reported poor early growth in HEU compared to HUU infants [27]. HEU infants may be at an increased risk of poor early growth due to immune activation [28] and systemic inflammation, due to in utero or postnatal exposure to HIV, as well as a prolonged exposure to ART [2,5,29]. Since all the PWLWH were on ART, we could not explore the latter association.

### 4.2. Altered Ketosis in Pregnant Mothers Living with HIV

Ketone bodies are small, water-soluble molecules produced in the liver comprising three molecules: 3-hydroxybutyric acid, acetoacetic acid, and acetone [30]. In response to the reduced availability of glucose, the body will increase the production of ketone bodies, called ketogenesis, as an alternative energy supply by breaking down fatty acids [31] and certain ketogenic amino acids. Ketone bodies are synthesized from acetyl-coenzyme A (acetyl-CoA), a product of the mitochondrial beta-oxidation of fatty acids. Acetyl-CoA can also be converted to acetic acid by acetyl-CoA hydrolase [32].

**Figure 1:**
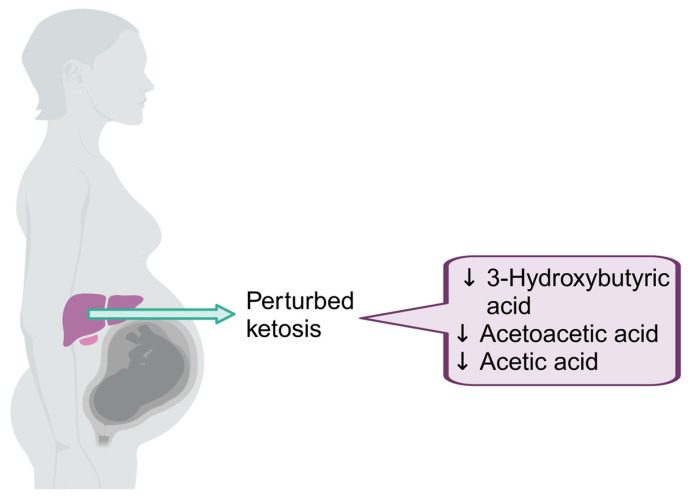
The metabolites differentially expressed in plasma in mothers living with HIV compared to controls. Three metabolites, 3-hydroxybutyric acid, acetoacetic acid, and acetic acid, were lower in the PWLWH compared to the HIV-uninfected mothers at 28 weeks’ gestation. Image created in BioRender.

In response to the needs of fetal growth and development, pregnant women undergo a series of changes concerning glucose metabolism. These changes include (1) an increased insulin resistance in surrounding tissues, (2) an increased utilization and excretion of glucose, (3) an increased gluconeogenesis in the liver during the third trimester, and (4) accelerated lipolysis [33]. In our cohort, the concentration of two ketone bodies, 3-hydroxybutyric acid and acetoacetic acid, were significantly lower in the PWLWH on ART compared to the pregnant women living without HIV. This indicates an impaired ketogenesis associated with HIV-ART exposure. This could be due to increased glucose utilization as a fuel source in the group living with HIV. It is well known that HIV and ART dysregulate normal glucose metabolism [34], accounting for the differences observed in the ketone body concentrations. Lower levels of plasma acetoacetic acid in PLWH, compared to healthy controls, have been reported before [35]; thus, our results confirm this observation. However, we also detected lower 3-hydroxybutyric acid and acetic acid levels, strengthening the finding that ketosis is negatively affected in PWLWH on ART (Figure 1).

### 4.3. Metabolic Profile of HEU Infants at Birth Linked to Compromised Glucose Metabolism with Possible Aberrant Neurological Development

In this cohort, the HEU infants had significantly lower levels of threonine and myo-inositol than the HUU infants at birth. Threonine is an essential amino acid while myo-inositol is a cyclic sugar molecule, synthesized de novo from glucose and through the catabolism of phosphatidylinositol, phosphoinositides, and inositol phosphates [36]. Lower plasma threonine levels have previously been associated with insulin resistance and metabolic syndrome, while lower plasma myo-inositol levels have been observed in individuals with insulin resistance, metabolic syndrome, and gestational diabetes [37]. Therefore, the lower threonine and myo-inositol levels detected in the HEU possibly indicate perturbed glucose metabolism. Since the PWLWH had altered ketosis at 28 weeks’ gestation (indicated by lower 3-hydroxybutyric acid, acetoacetic acid, and acetic acid levels), probably indicating glucose metabolism perturbations, the perturbed glucose metabolism detected in the HEU can probably be attributed to the metabolic perturbation of the mothers during the last trimester. Nevertheless, perturbed glucose metabolism in infancy can impact brain development [38], overall growth [39], and metabolic health [40], which may play a role in the significantly lower BMI (*p* = 0.0261) detected in the HEU compared to the HUU infants at birth.

Formic acid and its conjugate base, formate, are endogenous, essential metabolites found in almost all living organisms. They are simple one-carbon metabolites at the heart of the folate-mediated one-carbon metabolism cycle [41,42]. One-carbon metabolism is a fundamental cellular process that supports multiple physiological functions and is essential for cellular homeostasis, growth, and development. Therefore, formic acid is a valuable biomarker of metabolic impairment involving vitamin B12 deficiencies [43]. At birth, the formic acid concentration was higher in the HEU infants than in the HUU infants. Elevated formic acid levels have previously been reported in PLWH compared to healthy controls [44]. Kavitha et al. [44] reported that formic acid levels increased further following ART administration, but did not elaborate on reasons for the elevated formic acid levels. A possible explanation for this observation could be lowered vitamin B12 levels since (i) it is known that elevated formic acid is a potential biomarker for vitamin B12 deficiency [43] and (ii) ART lowers vitamin B12 levels [35]. With this in mind, the ART of the PWLWH could potentially cause the higher formic acid levels detected in the HEU. Nonetheless, the consequence of higher formic acid levels should be a reason for concern in the HEU since formic acid is a toxic metabolite, and the elevation of its concentration during folate and vitamin B12 deficiencies may elicit some of the pathologies (e.g., neurocognitive and neuropsychiatric) associated with vitamin B12 deficiencies [43]. This notion could lead to neurological abnormalities in these infants. Taken together with the lower plasma myo-inositol and threonine levels in the HEU infants, with the possible link of the latter to brain development in the infant, clearly raises concerns about the risk of these infants for neurological aberrations and warrants further investigation.

### 4.4. Risk of Increased Inflammation in HEU at 6–10 Weeks and Altered Glucose Metabolism at 6 Months Post-Partum

At 6–10 weeks post-partum, the HEU infants had lower plasma tyrosine and betaine levels. Betaine, a metabolite found in animals, plants, and microorganisms, is mainly synthesized via the catabolism of choline in the one-carbon metabolism, and has been shown to play an anti-inflammatory role in humans [45]. In our cohort, betaine concentrations were lower in the HEU infants than in the HUU infants. HEU infants are known to have increased inflammation at birth, which persists for the first six months of life [28]. Since betaine plays an anti-inflammatory role, the decrease in betaine levels in the HEU group is consistent with the literature and could be one of the contributing factors to the theorized persistence of inflammation in these infants.

Tyrosine is a non-essential amino acid that is readily synthesized from phenylalanine. Its main function is proteinogenic (i.e., it is used in the biosynthesis of proteins) [46]. A study examining the phenylalanine to tyrosine ratio in PLWH [47] found that there was a decrease in the level of tyrosine, which may relate to a diminished conversion of phenylalanine to tyrosine via the enzyme phenylalanine-hydroxylase. This phenomenon is commonly seen in PLWH and is related to increased immune activation markers, e.g., neopterin or interferon-gamma [48,49]. This subsequent increase in immune activation markers has been linked to an increase in infections experienced by PLWH [50]. The lower levels of tyrosine in HEU infants, compared to that of HUU infants detected in our cohort could be due to exposure to HIV in utero. While the link between these metabolic alterations and immune function in the infants in our study is currently speculative, further work is ongoing to test this hypothesis.

At six months of age, HEU infants had lower 3-hydroxyisobutyric acid (3-HIBA) levels and higher glycine levels compared to the HUU infants. 3-HIBA is a catabolic product of valine, a branched-chain amino acid. Elevated 3-HIBA levels are indicative of mitochondrial dysfunction, since branched-chain metabolism is halted in respiratory chain deficiencies [51]. However, the lower 3-HIBA levels detected in the HEU might indicate higher valine catabolism. The exact reason for this remains unclear, but altered branch-chain metabolism is often linked to perturbed glucose metabolism [51].

**Figure 2:**
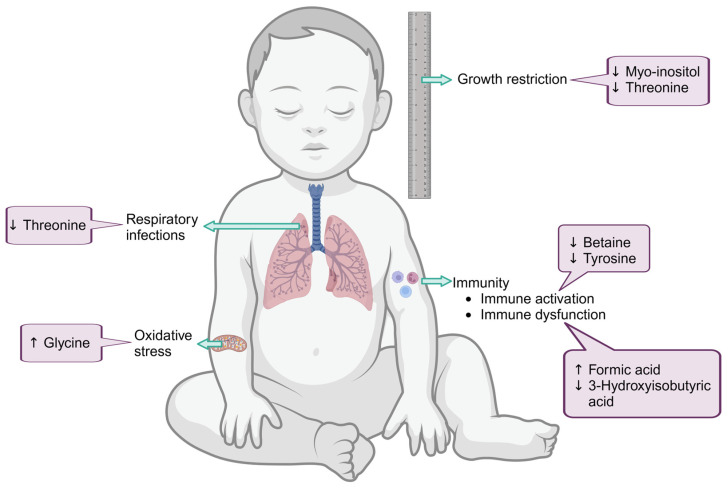
Metabolites are differentially expressed in plasma HEU and HUU infants. In infants at birth, threonine and myo-inositol levels were lower in the HEU group and formic acid levels were higher. At 6 or 10 weeks, betaine and tyrosine levels were lower in the HEU group. Finally, at six months, 3-hydroxyisobutyric acid levels were lower, while glycine levels were higher in the HEU infants. The figure also indicates the potential effects of the metabolite differences on the systemic functions of the study participants. Image created in BioRender.

Glycine is the smallest amino acid in nature and is involved in metabolic regulation, antioxidant reactions, and neurological function [52]. Glycine plays a crucial role in the synthesis of glutathione, a key antioxidant that helps protect cells from oxidative damage. The higher urinary glycine levels detected in the HEU infants might indicate upregulated glutathione synthesis. This aspect warrants further investigation in HEU infants, since it is well known that viral infections lead to increased oxidative stress [53] (Figure 2).

The findings of this study clearly demonstrate the potential for the development of several pathologic conditions based on the metabolic abnormalities observed. This begs the question: what can be done to compensate for these abnormalities? One of the most significant adjustments that can be made in the HEU group is to advocate for continued breastfeeding until six months post-partum, since all the metabolites found to be decreased in the HEU group naturally occur in breastmilk [54,55,56,57,58]. Our data clearly demonstrate a lower frequency of breastfeeding in the HEU group compared to the HUU group, which could account for some of the metabolite differences observed. The reality is that breastfeeding in MLWH is complicated by the fear of transmitting the virus to their children, as well as limitations on the duration of breastfeeding secondary to socio-economic factors, especially in resource-limited settings. Risk-stratified counseling should be explored in these settings where breastfeeding is strongly recommended. Other therapies and supplementation strategies to correct metabolic derangements have not been explored in HEU infants and should be considered in future nutrimetabolomic [59] studies.

A clear limitation of our study is the use of only one biofluid. Further studies on urine and possibly saliva could provide a more comprehensive overview of the metabolome in these groups. Another limitation is the use of NMR. While it provides us with high repeatability as well as the added advantage of being cost-effective and utilizing only a small sample volume, it does not provide the ability to screen for larger, more complex biomolecules that other technologies such as gas chromatography–mass spectrometry or liquid chromatography–mass spectrometry would provide. The small sample size also precluded the use of more advanced statistical analysis such as multivariable regression analysis.

Our study does also have significant strengths, such as the use of a socio-culturally matched control group and the use of a single ART regimen in all PWLWH, as well as the longitudinal nature of the study.

## 5. Conclusions

Our data suggest that the in utero exposure to HIV/ART causes a difference in some key metabolites observed in pregnant women and their infants at various time points post-partum. These metabolites are particularly important in energy utilization and protection from infection. Strategies to optimize breastfeeding and improve nutritional practices in HEU may improve the long-term growth outcomes in this growing and vulnerable population. Future studies should investigate the importance of these metabolites in larger populations.

## Figures and Tables

**Table 1:** Demographic and clinical characteristics of mothers with and without HIV at 28 weeks’ gestation and at birth.

Time Point	28 Weeks’ Gestation			Birth		
Group	PWLWH (*n* = 29)	HIV-Uninfected (*n* = 31)	*p*-Value	MLWH (*n* = 29)	HIV-Uninfected (*n* = 31)	*p*-Value
Age at time of delivery (years)				37.0 (32.0–39.0)	28.0 (24.0–36.0)	0.0021
CD4+ T-cell count (cells/µL)	317.5 (220.0–538.0)			351.0 (220.0–587.0)		
Viral load (copies/mL)	143.0 (40.0–243.0)			150.0 (40.0–217.0)		
Weight (kg)	72.5 ± 12.4	68.7 ± 10.5	0.1851	69.9 ± 12.1	71.6 ± 11.3	0.7081
Height (cm)	160.2 ± 6.0	158.1 ± 6.0	0.1383	160.2 ± 6.0	158.1 ± 6.0	0.1383
BMI (kg/m^2^)	28.1 ± 4.0	27.6 ± 4.7	0.5227	27.3 ± 4.0	28.6 ± 5.1	0.3682
MUAC (cm)	29.6 ± 4.2	27.5 ± 3.6	0.0112	29.1 ± 4.4	29.1 ± 5.0	0.9515
Delivery method				10 (36%) C/S 18 (64%) NVD 1 information missing	18 (58%) C/S 13 (42%) NVD	0.1330

All results are shown as median values with interquartile ranges (IQRs) for skewed distributions or mean values ± standard deviation (SD) for normal distributions. Where cells are shaded, the parameters are not applicable to the group. Abbreviations—PWLWH: pregnant women living with HIV, MLWH: mothers living with HIV, kg: kilograms, cm: centimeters, BMI: body mass index, MUAC: mid-upper-arm circumference, C/S: caesarean section, NVD: normal vaginal delivery.

**Table 2:** Demographic and clinical characteristics of infants exposed and unexposed to HIV at birth and six weeks.

Time Point	Birth			6 Weeks		
Group	HEU (n = 29)	HUU (n = 31)	*p*-Value	HEU (n = 29)	HUU (n = 31)	*p*-Value
Weight (kg)	2.9 ± 0.5	3.0 ± 0.4	0.5199	4.3 (3.8–4.7)	4.6 (4.1–4.9)	0.0375
Length (cm)	50.0 ± 3.9	49.7 ± 3.4	0.6348	54.5 (52.6–55.6)	53.7 (52.5–56.0)	0.8673
BMI (kg/m^2^)	11.5 ± 1.6	12.1 ± 1.0	0.0261	14.2 ± 1.9	15.6 ± 1.7	0.0045
HC (cm)	34.4 ± 2.0	34.0 ± 1.7	0.4380	37.9 (36.7–38.5)	38.0 (37.4–39.1)	0.1275
MUAC				12.9 (11.9–13.1)	13.3 (12.7–14.0)	0.0612
Weight z-score	−0.6 ± 1.4	−0.3 ± 1.1	0.4915			
Length z-score	0.7 ± 2.1	0.5 ± 1.9	0.5383			
HC z-score	0.7 (−0.2–1.8)	0.6 (−0.2–1.3)	0.4030			
WHZ				−0.5 (−1.4–0.6)	0.4 (−0.3–1.7)	0.0123
WAZ				−0.6 ± 1.7	0.19 ± 1.4	0.0539
HAZ				−0.4 (−1.5–1.2)	−0.4 (−1.2–0.5)	0.9033
BAZ				−0.5 (−1.2–0.5)	0.7 (−0.2–1.1)	0.0096
HCZ				0.5 (−0.4–1.5)	0.8 (0.1–1.5)	0.2519
Breastfeeding	27/29 (93.1%)	31/31 100%	0.2290	21/27 (77.8%)	30/31 (96.8%)	0.0330

All results are shown as median values with interquartile ranges (IQRs) for skewed distributions or mean values ± standard deviation (SD) for normal distributions, except for breastfeeding status, presented as percentages (%). Where cells are shaded, the parameters are not applicable to the group. Abbreviations—HEU: HIV-exposed uninfected, HUU: HIV-unexposed uninfected, kg: kilograms, cm: centimeters, BMI: body mass index, HC: head circumference, MUAC: mid-upper-arm circumference, WHZ: weight-for-length z-score, WAZ: weight-for-age z-score, HAZ: height-for-age z-score, BAZ: BMI-for-age z-score, HCZ: head circumference z-score.

**Table 3:** Demographic and clinical characteristics of infants exposed and unexposed to HIV at 10 weeks and 6 months.

Time Point	10 Weeks			6 Months		
Group	HEU (n = 29)	HUU (n = 31)	*p*-Value	HEU (n = 29)	HUU (n = 31)	*p*-Value
Weight (kg)	5.3 (4.8–5.7)	5.6 (5.0–5.9)	0.1867	7.1 (6.8–7.6)	7.4 (6.8–8.0)	0.2454
Length (cm)	58.5 (56.4–59.7)	57.3 (56.4–58.8)	0.2841	66.4 (64.1–68.0)	66.5 (64.0–68.8)	0.6233
BMI (kg/m^2^)	15.2 ± 2.1	16.9 ± 2.2	0.0136	16.6 ± 1.1	17.2 ± 2.0	0.3456
HC (cm)	39.3 (39.0–40.4)	39.6 (39.0–40.2)	0.9362	44.0 (43.0–44.6)	43.1 (42.4–44.0)	0.1155
MUAC	13.8 (13.0–14.5)	13.8 (13.0–15.0)	0.6518	14.2 (14.0–15.0)	15.0 (14.0–16.0)	0.0465
WHZ	−0.4 (−1.2–0.8)	0.6 (−0.6–1.4)	0.0190	−0.2 (−0.7–0.4)	0 (−0.8–0.8)	0.4086
WAZ	−0.5 ± 1.4	0.04 ± 1.1	0.1335	−0.7 ± 0.9	−0.2 ± 1.3	0.1101
HAZ	0.2 (−1.3–0.9)	−0.4 (−1.1–0.7)	0.4335	−0.3 (−1.5–0.5)	−0.5 (−1.1–0.8)	0.3416
BAZ	−0.5 (−1.0–0.2)	0.7 (−0.6–1.0)	0.0053	−0.4 (−0.8–0.1)	−0.1 (−1.0–0.7)	0.3577
HCZ	0.7 (−0.2–1.5)	0.6 (0.1–1.2)	0.9745	0.7 (−0.2–1.1)	0.5 (−0.4–1.0)	0.3869
MUACZ				0.2 (−0.2–0.7)	0.7 (0.2–1.5)	0.0381
Breastfeeding	18/28 (64.3%)	30/31 (96.8%)	0.0020	16/28 (57.1%)	22/31 (71.0%)	0.1680

All results are shown as median values with interquartile ranges (IQRs) for skewed distributions or mean values ± standard deviation (SD) for normal distributions, except for breastfeeding status, presented as percentages (%). Where cells are shaded, the parameters are not applicable to the group. Abbreviations—HEU: HIV-exposed uninfected, HUU: HIV-unexposed uninfected, kg: kilograms, cm: centimeters, BMI: body mass index, HC: head circumference, MUAC: mid-upper-arm circumference, WAZ: weight-for-age z-score, LAZ: length-for-age z-score, WHZ: weight-for-length z-score, HAZ: height-for-age z-score, BAZ: BMI-for-age z-score, HCZ: head circumference z-score, MUACZ: mid-upper-arm circumference z-score.

**Table 4:** Metabolites of significance that differed between the study groups.

Time Point	Metabolite	PWLWH/HEU (n = 29)	HIV-Uninfected/HUU (n = 31)	*p*-Value
Mothers at 28 weeks’ gestation	3-Hydroxybutyric acid	43.8 (28.5–59.3)	61.8 (41.8–118.8)	0.0351
	Acetoacetic acid	17.6 (13.2–23.2)	25.6 (15.1–58.7)	0.0241
	Acetic acid	34.7 (23.6–48.3)	44.9 (29.8–54.9)	0.0377
Infants at Birth	Threonine	218.3 (170.9–277.5)	293.5 (245.4–351.3)	0.0076
	Myo-inositol	196.7 (151.9–238.0)	252.0 (193.2–307.6)	0.0500
	Formic acid	32.8 (16.8–52.1)	19.6 (13.2–24.7)	0.0500
Infants at 6/10 weeks post-partum	Betaine	143.0 (108.2–164.3)	162.5 (148.1–213.6)	0.0176
	Tyrosine	94.9 (76.9–135.9)	134.1 (94.4–151.7)	0.0483
Infants at 6 months post-partum	3-Hydroxyisobutyric acid	12.7 (6.9–14.3)	15.3 (7.1–18.2)	0.0390
	Glycine	324.6 (292.6–371.3)	264.3 (230.6–323.3)	0.0078
**Excluding infants not breastfed**				
Infants at 6/10 weeks post-partum	Betaine	147.6 (119.0–193.7)	166.1 (148.6–213.6)	0.0453
	Tyrosine	94.5 (74.6–125.0)	132.3 (94.4–144.0)	0.0431
	Glycine	332.3 (290.2–362.8)	283.9 (239.3–348.0)	0.0315
Infants at 6 months post-partum	3-Hydroxyisobutyric acid	12.0 (6.2–14.8)	13.4 (4.8–17.9)	0.4643
	Glycine	329.6 (297.2–378.8)	265.8 (233.2–323.3)	0.0205

Plasma metabolites differed significantly between mothers living with HIV and their HEU infants compared to HIV-uninfected mothers and their HUU infants. All results are shown as median values with interquartile ranges (IQRs) for skewed distributions. Abbreviations—PWLWH: pregnant women living with HIV, HEU: HIV-exposed uninfected, HUU: HIV-unexposed uninfected.

## Data Availability

The data presented in this study are available on request from the corresponding author. The data are not publicly available due to the privacy of the participants.

## Supplementary Materials

The following supporting information can be downloaded at: https://www.mdpi.com/article/10.3390/v16020313/s1, Table S1: Demographic and clinical characteristics of infants exposed and unexposed to HIV at 6 and 10 weeks and 6 months; Table S2: Metabolomics data of pregnant women living with HIV and HIV-uninfected pregnant women at 28 weeks’ gestation; Table S3: Metabolomics data of infants exposed and unexposed to HIV at birth; Table S4: Metabolomics data of infants exposed and unexposed to HIV at 6/10 weeks; Table S5: Metabolomics data of infants exposed and unexposed to HIV at 6 months.
