# Supplementary material for: Metabolic Alterations in Mothers Living with HIV and Their HIV-Exposed, Uninfected Infants
Source: Viruses. 2024 Feb 19;16(2):313. doi: 10.3390/v16020313 (PMC10892778; doi:10.3390/v16020313)
Supplement: Supplementary file 1 [file viruses-16-00313-s001.zip › viruses-2775289-supplementary.pdf]

Supplementary Information

Supplementary Table S1: Demographic and clinical characteristics of infants exposed and unexposed to HIV at 6 and 10 weeks and 6 months. These data demonstrate the sensitivity analysis of infants who were exclusively breastfed at these time points.

Time point	6 weeks			10 weeks			6 months		
**Group**	**HEU (n = 21)**	**HUU (n = 30)**	**p-value**	**HEU (n = 18)**	**HUU (n= 30)**	**p-value**	**HEU (n = 16)**	**HUU (n = 22)**	**p-value**
Weight (kg)	4.1 (3.8 – 4.7)	4.6 (4.1 – 4.9)	**0.0386**	5.1 (4.8 – 5.7)	5.4 (4.9 – 5.8)	0.2511	7.0 (6.7 – 7.6)	7.4 (7.0 – 7.8)	0.1628
Length (cm)	54.3 (52.6 – 55.5)	53.6 (52.5 – 56.0)	0.8406	59.2 (56.0 – 60.0)	57.2 (56.4 – 58.6)	0.2275	65.4 (62.4 – 68.0)	66.4 (64.0 – 68.0)	0.5699
BMI (kg/m^2)	13.9 ±2.1	15.5 ±1.6	**0.0083**	15.1 ±2.1	16.6 ±2.1	**0.0246**	16.6 ±0.9	17.2 ±1.9	0.3144
HC (cm)	37.8 (36.8 – 38.3)	38.0 (37.4 – 39.0)	0.0894	39.1 (39.0 – 40.0)	39.6 (39.0 – 40.2)	0.7321	44.0 (43.0 – 44.0)	43.0 (42.0 – 44.0)	0.2732
MUAC	12.9 (12.3 – 13.0)	13.0 (12.7 – 14.0)	0.1161	13.8 (13.0 – 15.0)	13.7 (13.0 – 15.0)	0.9049	14.0 (14.0 – 14.2)	15.0 (14.0 – 16.0)	**0.0125**
WHZ	-0.7 (-1.7 – 0.7)	0.4 (-0.3 – 1.7)	**0.0186**	-0.4 (-1.0 – 0.3)	0.5 ±1.9	**0.0303**	-0.1 (-0.6 – 0.3)	0.0 (-0.6 – 0.6)	0.4171
WAZ	-0.8 ±1.7	0.1 ±1.3	0.0607	-0.7 ±1.5	0.0 (-0.6 – 0.6)	0.1466	-0.8 ±0.9	-0.2 ±1.0	0.0717
HAZ	-0.4 (-1.5 – 0.9)	-0.4 (-1.2 – 0.1)	0.9694	0.2 (-1.3 – 0.9)	-0.5 (-1.1 – 0.4)	0.5239	-0.9 (-2.1 – 0.6)	-0.5 (-1.0 – 0.7)	0.4171
BAZ	-0.8 (-1.6 – 0.4)	0.6 (-0.2 – 1.0)	**0.0163**	-0.5 (-0.9 – 0.1)	0.6 (-0.7 – 0.9)	**0.0154**	-0.3 (-0.8 – 0.1)	-0.0 (-0.7 – 0.5)	0.3144
HCZ	0.5 (-0.4 – 1.5)	0.7 (0.1 - 1.5)	0.1980	0.5 (-0.4 – 1.1)	0.5 (0.1 – 1.1)	0.6406	0.6 (-0.3 – 0.9)	0.5 (-0.7 – 0.6)	0.4752
MUACZ							0.1 (-0.2 – 0.3)	1.0 (0.2 - 1.8)	**0.0155**

All results are shown as median values with interquartile ranges (IQRs) for skewed distributions or mean values with ± standard deviation (SD) for normal distributions. Where cells are shaded, the parameters are not applicable to the group. Abbreviations: HEU: HIV-exposed uninfected, HUU: HIV-unexposed uninfected, kg: kilograms, cm: centimeters, BMI: body mass index, HC: head circumference, MUAC: mid-upper arm circumference, WHZ: weight-for-length z-score, WAZ: weight-for-age z-score, HAZ: height-for-age z-score, BAZ: BMI-for-age z-score, HCZ: head circumference z-score, MUACZ: mid-upper arm circumference z-score.

Supplementary Table S2: Metabolomics data of pregnant women living with HIV and HIV-uninfected pregnant women at 28 weeks' gestation.

Time point	28 weeks' gestation		
**Group**	**PWLWH (n = 29)**	**HIV-uninfected (n = 31)**	**p-value**
3-hydroxybutyric acid	43.8 (28.5 – 59.3)	61.8 (41.8 – 118.8)	**0.0351**
3-Hydroxyisobutyric acid	8.6 (5.4 – 11.8)	10.8 (6.8 – 14.3)	0.2007
3-Hydroxyisovaleric acid	4.3 (2.8 – 5.6)	4.2 (3.1 – 6.4)	0.6733
Acetic acid	34.7 (23.6 – 48.3)	44.9 (29.8 – 54.9)	**0.0377**
Acetoacetic acid	17.6 (13.2 – 23.2)	25.6 (15.1 – 58.7)	**0.0241**
Acetone	3.2 (2.3 – 5.4)	5.5 (2.2 – 10.5)	0.0848
Betaine	93.1 (81.6 – 113.3)	94.9 (79.6 – 105.0)	0.6733
CA-EDTA	2682.1 (2384.2 – 2880.9)	2694.2 (2440.6 – 2944.9)	0.7393
Creatinine	46.0 (36.4 – 57.9)	50.7 (39.9 – 58.2)	0.3708
Formic acid	23.1 (15.2 – 40.1)	22.4 (15.3 – 32.6)	0.8186
Glucose	1606.3 (1274.7 – 1904.2)	1468.4 (1298.8 – 1762.8)	0.5104
Glycine	226.4 (181.1 – 278.3)	228.4 (176.3 – 253.8)	0.7730
Hypoxanthine	12.8 (7.6 – 18.1)	7.4 (4.1 – 21.8)	0.1451
Myo-inositol	92.1 (63.6 – 113.8)	85.2 (58.5 – 118.0)	0.9705
Taurine	1794.1 (1526.7 – 2009.1)	1688.8 (1524.0 -1982.1)	0.5296
Threonine	190.0 (79.4 – 253.5)	173.6 (119.0 – 219.2)	0.8072
Tyrosine	45.4 (25.1 – 61.3)	44.8 (33.5 – 57.9)	0.9943
Valine	126.5 (107.3 – 162.8)	136.8 (120.7 – 174.2)	0.1905

All results are shown as median values with interquartile ranges (IQRs) for skewed distributions. Abbreviations: PWLWH: pregnant women living with HIV.

Supplementary Table S3: Metabolomics data of infants exposed and unexposed to HIV at birth.

**Group**	**HEU (n = 29)**	**HUU (n = 31)**	**p-value**
3-Hydroxybutyric acid	235.9 (136.5 – 440.6)	217.7 (118.9 – 307.5)	0.5645
3-Hydroxyisobutyric acid	15.8 (12.4 – 25.3)	16.3 (9.9 – 22.0)	0.5748
3-Hydroxyisovaleric acid	3.9 (12.4 – 25.3)	5.5 (2.6 – 7.9)	0.2684
Acetic acid	44.2 (38.9 – 49.8)	47.3 (38.7 – 55.9)	0.2619
Acetoacetic acid	24.9 (18.5 – 50.9)	26.4 (18.8 – 43.0)	0.6934
Acetone	21.6 (6.7 – 43.6)	12.0 (7.4 – 17.7)	0.3710
Betaine	91.4 (80.7 – 106.6)	98.1 (78.4 – 116.9)	0.3472
CA-EDTA	2783.3 (2561.7 – 2915.0)	2706.8 (2435.3 – 3164.5)	0.6062
Creatinine	77.5 (64.5 – 112.9)	91.0 (76.6 – 119.0)	0.1541
Formic acid	32.8 (16.8 – 52.1)	19.6 (13.2 – 24.7)	**0.0500**
Glucose	948.6 (635.1 – 1450.7)	1035.2 (709.5 – 1374.2)	0.4043
Glycine	325.4 (279.9 – 391.7)	363.1 (295.8 – 460.6)	0.2555
Hypoxanthine	13.5 (9.6 – 23.0)	17.3 (9.8 – 26.9)	0.4043
Myo-inositol	196.7 (151.9 – 238.0)	252.0 (193.2 – 307.6)	**0.0500**
Taurine	1343.6 (923.2 – 1680.9)	1570.3 (1014.1 – 1810.0)	0.2885
Threonine	218.3 (170.9 – 277.5)	293.5 (245.4 – 351.3)	**0.0076**
Tyrosine	79.4 (65.0 – 90.7)	78.0 (61.5 – 99.6)	0.6062
Valine	217.2 (178.2 – 248.4)	223.8 (187.2 -262.3)	0.4304

All results are shown as median values with interquartile ranges (IQRs) for skewed distributions. Abbreviations: HEU: HIV-exposed uninfected, HUU: HIV-unexposed uninfected.

Supplementary Table S4: Metabolomics data of infants exposed and unexposed to HIV at 6/10 weeks.

**Group**	**HEU**<br>**(n = 29)**	**HUU**<br>**(n = 31)**	**p-value**
3-Hydroxybutyric acid	61.5 (42.1 – 77.6)	62.9 (51.4 – 90.7)	0.5491
3-Hydroxyisobutyric acid	14.0 (10.6 – 15.6)	12.2 (6.7 – 15.3)	0.3253
3-Hydroxyisovaleric acid	3.6 (2.1 – 5.1)	2.8 (1.5 – 4.3)	0.4640
Acetic acid	48.5 (37.2 – 65.4)	54.3 (44.2 – 78.4)	0.4374
Acetoacetic acid	16.7 (12.8 – 22.1)	23.0 (12.3 – 27.8)	0.2769
Acetone	5.5 (3.9 – 10.7)	5.2 (3.2 – 9.8)	0.4374
Betaine	143.0 (108.2 – 164.3)	162.5 (148.1 – 213.6)	**0.0176**
CA-EDTA	2582.1 (2383.6 – 2850.2)	2801.8 (2490.5 – 3093.1)	0.1186
Creatinine	91.7 (75.4 – 116.5)	98.2 (74.3 – 113.7)	0.9117
Formic acid	97.7 (59.3 – 143.9)	90.5 (57.9 – 121.2)	0.6626
Glucose	1550.3 (1270.9 – 1790.8)	1570.9 (1413.2 – 1973.7)	0.3868
Glycine	332.3 (290.2 – 362.8)	286.3 (239.3 – 348.0)	0.0723
Hypoxanthine	12.6 (6.1 – 20.4)	14.9 (9.7 – 20.3)	0.7843
Myo-inositol	212.7 (131.5 – 262.6)	217.6 (154.9 – 275.3)	0.4287
Taurine	1878.7 (1659.2 – 2129.0)	2049.9 (1714.4 – 2281.3)	0.2970
Threonine	216.6 (135.6 – 279.1)	203.0 (127.9 – 279.6)	0.8186
Tyrosine	94.9 (76.9 – 135.9)	134.1 (94.4 – 151.7)	**0.0483**
Valine	178.5 (141.1 – 216.5)	196.2 (151.3 – 224.3)	0.6842

All results are shown as median values with interquartile ranges (IQRs) for skewed distributions. Abbreviations: HEU: HIV-exposed uninfected, HUU: HIV-unexposed uninfected.

Supplementary Table S5: Metabolomics data of infants exposed and unexposed to HIV at 6 months.

**Group**	**HEU (n = 29)**	**HUU (n = 31)**	**p-value**
3-Hydroxybutyric acid	79.9 (60.2 – 129.6)	86.0 (71.1 – 190.8)	0.3065
3-hydroxyisobutyric acid	12.7 (6.9 – 14.3)	15.3 (7.1 – 18.2)	**0.0390**
3-Hydroxyisovaleric acid	4.0 (1.7 – 6.0)	4.2 (2.6 – 6.2)	0.3645
Acetic acid	44.3 (35.5 – 54.4)	39.1 (30.6 – 51.6)	0.5758
Acetoacetic acid	18.9 (12.6 – 24.4)	19.3 (14.7 – 24.7)	0.6854
Acetone	5.8 (3.2 – 7.4)	9.0 (2.4 – 16.6)	0.1534
Betaine	154.6 (92.2 – 200.4)	164.5 (123.0 – 212.6)	0.3853
CA-EDTA	2868.8 (2497.7 – 3023.7)	2681.9 (2564.5 – 3142.1)	0.6296
Creatinine	101.0 (76.0 – 120.3)	102.5 (81.7 – 120.1)	0.8020
Formic acid	66.6 (38.6 – 90.4)	60.2 (41.5 – 102.4)	0.9079
Glucose	1366.5 (1025.9 – 1717.6)	1480.4 (1351.4 – 1730.3)	0.4286
Glycine	324.6 (292.6 – 371.3)	264.3 (230.6 – 323.3)	**0.0078**
Hypoxanthine	12.6 (9.5 – 25.1)	11.8 (7.0 – 17.0)	0.3960
Myo-inositol	133.9 (96.3 – 222.6)	130.1 (104.7 – 166.2)	0.7723
Taurine	1690.4 (1324.6 – 2117.1)	1775.1 (1551.1 – 2114.3)	0.6854
Threonine	194.3 (134.0 – 303.8)	194.0 (135.7 – 283.7)	0.6572
Tyrosine	81.1 (67.5 – 100.8)	82.7 (62.8 – 123.4)	0.8621
Valine	167.3 (134.7 – 229.7)	197.6 (143.8 – 246.0)	0.6296

All results are shown as median values with interquartile ranges (IQRs) for skewed distributions. Abbreviations: HEU: HIV-exposed uninfected, HUU: HIV-unexposed uninfected.
